# Pupillary reactivity to alcohol cues as a predictive biomarker of alcohol relapse following treatment in a pilot study

**DOI:** 10.1007/s00213-018-5131-1

**Published:** 2019-01-03

**Authors:** Timo L. Kvamme, Mads Uffe Pedersen, Morten Overgaard, Kristine Rømer Thomsen, Valerie Voon

**Affiliations:** 1 Centre for Alcohol and Drug Research, School of Business and Social Sciences, Aarhus University, Aarhus, Denmark; 2 Department of Psychiatry, University of Cambridge, Addenbrookes Hospital, Level E4, Hills Road, Box 189, Cambridge, CB2 0QQ UK; 3 Cognitive Neuroscience Research Unit, CFIN/MindLab, Aarhus University, Aarhus, Denmark; 4 Behavioral and Clinical Neurosciences Institute, University of Cambridge, Cambridge, UK; 5 NIHR Biomedical Research Council University of Cambridge, Cambridge, UK

**Keywords:** Alcohol, Relapse, Predictive, Pupillary response, Craving

## Abstract

**Rationale:**

Identifying the predictors of relapse in detoxified alcohol-dependent patients is crucial for effective surveillance procedures and the optimization of treatment. Physiological measures such as functional MRI activity and heart rate variability have been shown as potential markers of relapse prediction.

**Objectives:**

Our aim was to assess differential pupillary reactions to alcohol-related cues as an objective physiological candidate predictor of relapse.

**Methods:**

We examined the relationship between cue-elicited pupillary reactions to alcohol stimuli and luminance-controlled neutral stimuli in 21 detoxified alcohol-dependent patients and subsequent relapse outcome at a 4-month follow-up.

**Results:**

Differential pupillary dilation to alcohol stimuli as compared to neutral stimuli at 150 to 250 ms after stimulus onset substantially improved the model prediction of relapse outcome (additional 27% of variance) beyond that achieved from five standardized questionnaires on alcohol craving, alcohol use, problematic use severity, depressive tendencies, and duration of abstinence (47% of variance). In contrast, alcohol craving did not improve relapse model prediction.

**Conclusions:**

This pilot study shows that alcohol-dependent patients with greater pupillary dilation to alcohol stimuli are more vulnerable to relapse, and that pupillometry presents as an important tool for addiction science.

## Introduction

Relapse is a major issue in addiction science and identifying the predictors of relapse is critical for effective interventions in substance use disorders (Rounsaville 1986; Allen et al. 1996). In alcohol dependence, successful abstinence alone has minor bearing on whether a full-blown relapse or minor setbacks (“slip-ups”) will occur following the cessation of treatment (Brandon et al. 2007; Heinz et al. 2009). Identifying predictors of the degree of subsequent relapse in detoxified alcoholics is therefore crucial for effective surveillance procedures and the optimization of treatment. Here, we focus on cue-elicited pupillary reactions to alcohol cues as a candidate biomarker of relapse.

A mechanism contributing to relapse is the exposure to stimuli (cues) that have been repeatedly paired with the rewarding effects of alcohol and have thus become conditioned cues that can evoke conditioned responses, leading to alcohol craving and potential alcohol consumption (Robinson and Berridge 2001; Everitt and Robbins 2005). The empirical link between subjective alcohol craving and consumption remains uncertain as some studies do not find a relation in heavy drinkers (Field et al. 2005; MacKillop et al. 2010) whereas others do observe a relationship (Field et al. 2004; Kvamme et al. 2018). The link between alcohol craving and relapse outcome is even more unclear with equal numbers of studies with and without a reported correlations (see Heinz et al. for review). One reason for the poor consistency of findings could be attributed to the subjective measure of the construct of craving being vulnerable to denial and social desirability effects which could become exacerbated in a clinical research setting (Babor et al. 1987; Embree and Whitehead 1993; Tiffany and Carter 1998; Del Boca and Darkes 2003; Zemore 2012).

Exposure to alcohol cues can also evoke a broad array of physiological reactions in the central and autonomic nervous system which are less susceptible to the influence of subjective recall of the individual being measured. Biometric measures such as fMRI (Grüsser et al. 2004; Sinha and Li 2007), heart rate variability (Garland et al. 2012), and even measures of olfactory-induced salivation (Rohsenow et al. 1994) have been shown to be predictive of alcohol relapse. For instance, one study showed that the craving detoxified alcoholics report during exposure to alcohol stimuli was not predictive of subsequent relapse, while neurophysiological activity measured using fMRI was predictive (Grüsser et al. 2004). Cue-elicited physiological responses to alcohol cues is thought to reflect autonomic arousal in addition to heightened cognitive information processing (Tiffany and Carter 1998).

The pupils are the apertures which allow light into the eyes and, therefore, dilate as a reflex to light fluctuations in the environment (Goldinger and Papesh 2012). However, multiple studies show differential pupillary dilation in response to arousing and cognitively taxing stimuli under constant light conditions (Kuchinke et al. 2007; Claisse et al. 2016; Finke et al. 2017; Vasquez-Rosati et al. 2017). Moreover, as pupil dilation is an indirect measure of norepinephrine (NE) released from the locus coeruleus, it serves as a proxy of neural activity which is potentially more cost-efficient relative to fMRI (Siegle et al. 2003; Aston-Jones and Cohen 2005).

In this pilot study, differential pupillary reactions to alcohol stimuli and neutral control images were assessed for their predictive value of subsequent alcohol relapse outcome. We hypothesized that greater pupillary dilation to alcohol images would add incremental predictive value of relapse risk beyond traditional questionnaires assessing prior alcohol use, psychological addiction symptomology, and depressive tendencies. Furthermore, we aimed to investigate an indirect measure of subjective craving as a predictor of relapse along with its relationship to standardized measures.

## Methods

### Participants

Twenty-one patients (mean age = 48.9 ± 9.7, 9 male) were recruited from one inpatient and one outpatient treatment center in Denmark. All patients were screened by expert clinicians at their respective treatment center and fulfilled ICD-10 diagnostic criteria for alcohol dependence as part of the inclusion criteria. The mean Alcohol Use Disorder Identification Test (AUDIT) (Saunders et al. 1993) scores, which track problematic alcohol use the year before initiation of treatment, were 28.9 ± 6.7, with a score greater than 20 being a useful cutoff point for considering further diagnosis for alcohol dependency (see Table 1 for details). One month prior to treatment, patients in the study reported having been intoxicated on average 5.3 ± 1.8 days/week with an average of 25.7 ± 15.9 units (UK) of alcohol per day.

**Table 1:** Inter-correlation of variables

	Mean [SD]	1	2	3	4	5	6	7	8	9	10
1. Relapse^a^	1.7 [0.8]	1.00									
2. Pupillary bias	− 0.0007 [0.03]	.64*	1.00								
3. Craving rating	2.9 [2.0]	− .18	− .28	1.00							
4. OCDS	8.9 [5.4**]**	− .02	− .17	.49*	1.00						
5. DAQ	2.7 [1.2]	− .26	− .41	.22	.30	1.00					
6. AUQ	139.5 [80.9]	.08	− .13	.46	.13	.05	1.00				
7. AUDIT	28.9 [6.7]	.06	− .24	.67*	.36	.25	.71*	1.00			
8. BDI	15.6 [9.3**]**	− .15	− .16	.19	.62*	.42	.002	.41	1.00		
9. Age	48.9 [9.7]	− .05	.19	− .37	− .28	.20	− .24	− .44*	− .30	1.00	
10. Gender	m/f 9/12	− .02	.20	− .57*	− .25	− .15	− .48*	− .51*	− .02	.23	1.00

Gender was coded as male = 0, female = 1

*OCDS*, Obsessive Compulsive Drinking Scale; *DAQ*, Desire for Alcohol Questionnaire; *AUQ*, Alcohol Use Questionnaire; *AUDIT*, Alcohol Use Disorder Identification Test; *BDI*, Becks Depression Inventory. *m*/*f*, male to female ratio

*p<0.05

^a^Missing data of three patients

Patients were required to have normal or corrected to normal vision. Patients were not excluded based on comorbid diagnoses or use of medication. All patients provided informed consent to participate in the study, which was approved by the local ethics committee. All patients were undergoing treatment and were told that they would be viewing alcohol-related cues while being asked about craving for alcohol and were urged take advice from their therapists if they felt distress due to participation in the study. Patients were paid 10 £ to take part in the initial testing which took place at the respective treatment center and 15 £ for the follow-up. Eighteen patients took part in the follow-up and were used for the following analyses. Patients reported having been detoxified for at least 1 week before testing (mean 10 ± 13 weeks). After providing informed consent and before being tested, patients were breathalyzed using a Lifeloc FC10 (Lifeloc Technologies, CO, USA). All patients had an undetectable breath alcohol level.

### Questionnaires

Patients completed questionnaires in the presence of a psychologist. Problematic use of alcohol was measured using the AUDIT, which consists of a 10-item questionnaire developed as a screening instrument for hazardous and harmful alcohol consumption. Patients were asked to provide answers relating to their drinking patterns 1 year before they initiated treatment at their respective treatment center*.* Previous alcohol use was measured using the Alcohol Use Questionnaire (AUQ), which assesses quantity of alcohol within the last month (Mehrabian and Russell 1978). Patients were asked to provide answers relating to their drinking patterns 1 month before they initiated treatment at their respective treatment center. Craving for alcohol was assessed with the Desires for Alcohol Questionnaire (DAQ) (Love et al. 1998), which is a 14-item questionnaire consisting of Likert scales with a range of 1 (low craving) to 7 (high craving). Obsessive thoughts about alcohol was measured using the Obsessive Compulsive Drinking Scale (OCDS) (Anton et al. 1995), a 14 item questionnaire consisting of Likert scales with a range of 1 to 5. Low scores indicate that a person has rare occurring thoughts about alcohol and a high degree of control over thoughts relating to alcohol while high scores relate to frequent thoughts about alcohol that have an intrusive and uncontrollable characteristic. Depressive tendencies were assessed with Becks Depression Inventory (BDI) (Beck et al. 1988).

### Pupillary cue-reactivity task and craving ratings

#### Apparatus

Pupillary responses to alcohol stimuli were measured using a cue-reactivity protocol with neutral control pictures. Patients participating in the study were seated in front of a 17-in monitor (1920 × 1080 resolution, 60 Hz), laptop computer. Eye movement and pupillary data were recorded non-intrusively by a Tobii × 2–60 Eye tracker integrated into the panels of the monitor. The × 2–60 system tracks eye gaze bilaterally (each 16.6 ms), using an infrared light reflected from the pupil and cornea. An initial 6-point calibration procedure using Tobii Studio software at 60-cm viewing distance ensured patients gaze was calibrated to the tracker.

#### Stimuli

The cue-reactivity task included two types of stimuli, i.e., (1) 48 Alcohol images obtained from the internet representing the alcohol choices typically available in Denmark and (2) 48 neutral images obtained through a standardized picture set (Blechert et al. 2014) that were matched approximately for color, size, form, and complexity to the alcohol images. Images subtended an approximal visual angle of 3.8° × 6.9°. Brightness was assessed using Bioconductor’s “EBImage” package in R (Pau et al. 2010). To ensure equiluminance, custom scripts were used to correct brightness across alcohol and neutral images (resulting *p* value of mean brightness difference was *p* = 0.96). Presentation of images was interspersed with alcohol images occurring after their matched neutral control image. Sequence of images was the same across all participants. The stimulation interface was custom-programmed in Python using PsychoPy (Version 1.84.2) (Peirce 2007).

#### Procedure

**Fig. 1:**
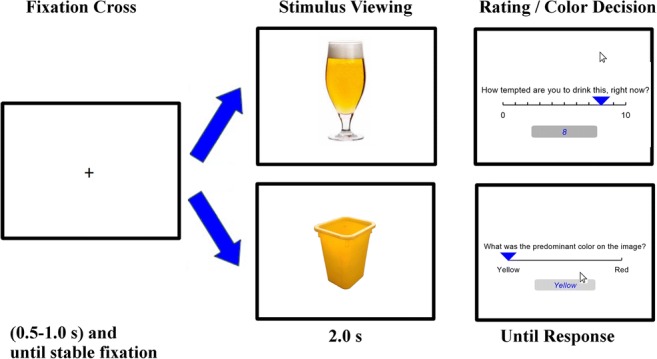
Cue-reactivity task. With separate task conditions of alcohol stimuli and neutral stimuli. Time courses are displayed in seconds (s). See “Methods” for details

Detailed instructions and practice trials were provided before the task commenced. Each trial (see Fig. 1) included three phases: (1) the fixation cross at the center image presented for a jittered 500–1000 ms, the duration of which was extended until eye positions without eye blinks were stable within 1.5° visual degrees of the fixation cross for a minimum of 200 ms. An instruction to fixate on the cross was displayed if this condition was not met within 2000 ms; (2) a stimulus picture of either alcohol or neutral was presented centrally for 2000 ms to which the patients were instructed to attend. Patients were allowed to “replay” the trial using a button press if they reported not having attended to the image. (3) Following neutral images, patients were asked which of two colors were predominant in the neutral object. The trial would get replayed upon an erroneous answer to the color decision. Following alcohol images, a rating slider from 0 to 10 was presented until the patient had provided a rating of how much they were tempted to drink the presented beverage. Patients took a mean of 3.3 s ± 1.2 s for the color discrimination and 3.9 ± 1.9 s for the craving ratings.

#### Craving scores instruction

Instruction for the rating of the alcohol images was very specific and was directly aimed at mitigating some of the previously noted difficulties in studying craving in a clinical environment. Patients were asked to rate their temptation to drink the specific alcohol shown under an imagined scenario where they were outside the clinical setting and would allow themselves to drink. Their preference of the alcohol was emphasized to avoid any underestimation of their ratings. We specifically wished to allow patients a choice that was free from the influence of the social desirability associated with being at a treatment center.

### Relapse rates

Four months following the cue-reactivity and questionnaire assessment, patients were contacted by phone by the same individual (TK) for information on their current relapse status (taking approximately 20 min). On the basis of the variance in the duration of relapse and the quantity of alcohol consumed during relapse, we opted to classify relapse into three categories of increasing severity of values (no relapse = 0, minor relapse = 1, major relapse = 2). Major relapse was defined as drinking persistently for more than a week as opposed minor relapse referring to any drinking (see (Reyes-Huerta et al. 2018) for a review on different relapse distinctions).

### Statistical analyses

Correlations were performed to evaluate the outcome in relapse and the relationship to potentially predictive variables and to investigate the convergent validity of craving ratings. Dependent on the distribution: the Pearson linear correlation coefficients were calculated between normally distributed variables, whereas Spearman rank correlations were calculated between non-normally distributed variables (relapse, craving rating, and gender).

In line with recommendations by Goldinger and Papesh (2012), pupil diameter values from both eyes measured during the cue-reactivity task were baseline-corrected by subtracting each sample of the 2000 ms stimulus viewing period from the 200-ms fixation phase immediately prior to stimulus presentation where fixation was deterministically stable. Only samples whose associated gaze positions were stable within the image dimensions were used for further analysis. Our fixation algorithm was based on the PyGaze detection algorithm (Dalmaijer et al. 2014) and was defined as gaze positions remaining stable within 1.5° visual degrees of the dimensions of the image for more than 60 ms. A chi-square test showed that samples not fitting these criteria were not predominantly from one stimulus type (*p* = 0.31). Outlying samples were rescaled using a Winsorizing procedure where values outside the 1.5 inter-quantile range (IQR) from the 25th to 75th percentile (the “Tukey Hinges”) of the distribution of values are rescaled to the outer valid value with the IQR (1.1% of the data) (Price et al. 2016). Pupillary bias scores were calculated for each patient for each sample by subtracting the mean baseline-corrected pupil diameter values recorded during presentation of alcohol images from those recorded during neutral images. For descriptive statistics, data were temporally smoothed using a moving average filter over a 100-ms time window.

To determine if relapse outcome at 4-month follow-up was related to pupillary bias, we used the Guthrie and Buchwald test (“gbtest”) from the “ERP” package freely available in the R programming language (Guthrie and Buchwald 1991; Sheu et al. 2016). This procedure tests whether the addition of relapse outcome adds significantly explained variance in the pupil diameter compared to the main effect of each patient for individual regression analyses performed at each sample in the waveform. The test corrects for multiple comparisons across the pupil waveform while considering the temporal autocorrelation of the data. Specifically, it holds type I error at *p* < 0.05 across the entire temporal window (in our case; 2000 ms) of the pupil waveform and performs Monte Carlo simulations (*n* = 1000) to empirically derive a subset of time points over which contiguous point-by-point correlations, each being significant at *p* < 0.05, can be considered jointly significant at *p* < 0.05 waveform-wise, given the temporal autocorrelation of the waveform. We used the entire 2000-ms time period as we did not have any *a priori* hypothesis about the temporal region of interest. The average diameter in pupillary bias for each patient during this “empirically derived window” was calculated for further analysis. Waveform analysis was also performed to test whether the neutral pupillary response alone was predictive of relapse and whether the pupillary bias co-varied with duration of abstinence.

### Multivariate analyses

To assess pupillary bias and craving rating’s predictive relationship with relapse outcome, hierarchical ordinal logistic regression modeling was carried out using Logit link functions. Relapse outcome was also assessed as a binary outcome of no relapse versus relapse (minor and major relapse combined). To control for other factors, the patients scores on questionnaires assessing psychological addiction symptomology (OCDS and DAQ), alcohol use (AUQ), severity of problematic alcohol use (AUDIT), and depressive tendencies (BDI) were entered in the first step. In the second step, we added either pupillary bias or craving rating separately to assess whether they were associated with relapse outcome beyond the aforementioned control variables. We also assessed whether baseline pupillary diameter in the 200-ms time window before stimulus onset and duration of abstinence was related to relapse outcome. All variables were X-standardized so that the coefficients indicate the increase of the dependent variable given an increase in the independent variables of one standard deviation (SD). Logit link was chosen because it allows the calculation of odds ratio for each coefficient through exponentiation of the model estimates. Positive odds ratio can be interpreted as how likely an increase in the ordinal dependent variable are for each unit SD increase in the covariate.

Model comparisons were performed using the differences in log-likelihood ratios between the first and the second step models including the added variable of interest. As this ratio approximates a *χ*^2^ distribution, it can be compared using a likelihood ratio chi-squared test (LR *χ*^2^) with the resulting *p* value assessing whether models differ significantly. Associated *p* values from the addition of either pupillary bias or craving rating were Bonferroni-corrected and considered significant at *p* < 0.05. To determine the difference in explained variance between models, we compared the likelihood ratio–based *R*^2^, which is a measure of fit analogous to the coefficient of determination (*R*^2^) in ordinary least square regression (Nagelkerke 1991). Unless specified, we report the more conservative adjusted *R*^2^, as this is more appropriate for models with small sample sizes and many fitted covariates (Heinzl and Mittlböck 2003). When estimating the total variation explained by a model, we compare the *R*^2^ to a null model, which only models the dependent variable with an intercept. Data analyses were performed using custom scripts in Python and R (Version 3.4.3) (R Core Team 2014).

## Results

### Relapse rates

Nine out of the 18 who took part in the follow-up (50%) reported being unable to remain abstinent over the course of the 4-month period following testing. We categorized five as having minor relapse (below 7 days). One patient reported having intended to drink only 2 units/week following treatment and had adhered to that goal. We categorized this as a minor relapse. Those in the minor relapse group had a mean of 2.7 ± 1.3 days relapse duration, (range 1–4), with 12.7 ± 7.3 units per mean day (range 5–20). Four were categorized as having a major relapse (above 7 days) over the course of or at the 4-month follow-up. Individuals in the major relapse group had a mean of 36.0 ± 22 days relapse duration (range 12–60) with 23.7 ± 17.3 units per mean day (range 7–48).

### Pupillary bias and relapse

**Fig. 2:**
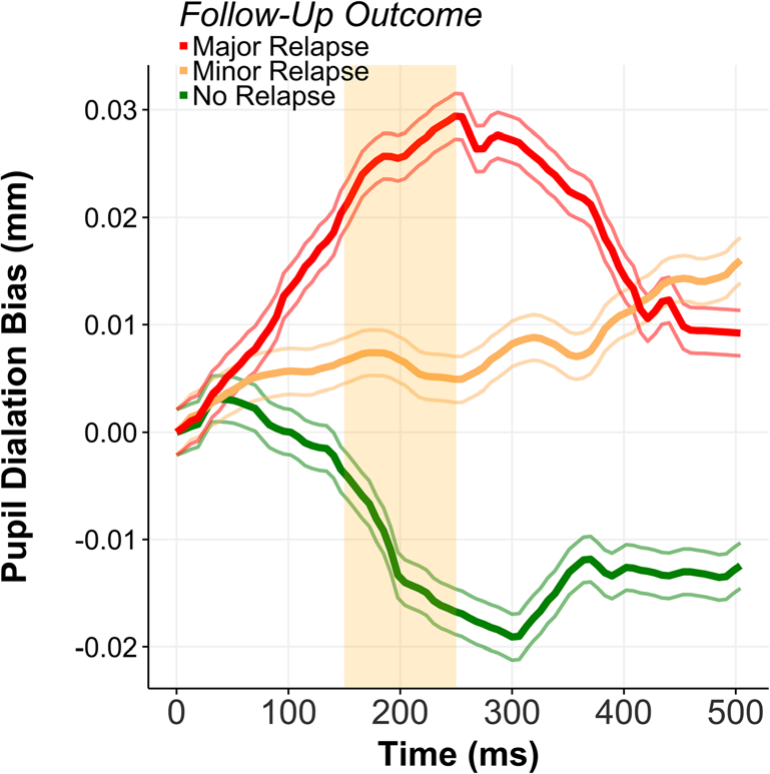
Pupillary diameter bias in millimeters (mm) for different relapse outcomes with positive values indicating greater extent of baseline-corrected pupil dilation on alcohol cues versus neutral across the time period. For clarity a subset of 500 milliseconds (ms) is shown. Orange-shaded squares indicate the empirically derived time window (derived from the entire 2000 ms) where pupillary bias is associated with relapse outcome above main effect of individual pupillary bias. Thin lines represent ± confidence intervals

Subsequent relapse outcome was associated with pupillary bias: in a waveform analysis, differences in pupillary dilation from 150 to 250 ms (7 samples) after stimulus onset was associated with an ordinal outcome measure of relapse rate above the main effect of patient’s individual pupil dilation differences (see Fig. 2). Patients showing a larger pupillary dilation during this time window to alcohol as compared to neutral had a higher likelihood of belonging to either the minor relapse or major relapse group. Furthermore, a waveform analysis using a dichotomous category of no relapse and relapse (minor and major collapsed into one) was similarly correlated with pupillary biases during the same 150–250-ms window. Which we confirmed with a point-biserial Pearson correlation between the dichotomous relapse variable and pupillary biases coefficient (*R* = 0.61, *p* = 0.007). Pupillary baseline in the neutral trials (200 ms before stimulus onset) did not drift over the course of the trials (*R* = − 0.258, *p* = 0.402). A waveform analysis using only pupillary responses to neutral stimuli showed no association with relapse outcome. We also confirmed by means of a waveform analysis that differences in pupillary dilation was not associated with duration of abstinence prior to the study.

### Univariate analyses

Correlations between potentially confounding demographic factors, clinical factors, and the relapse outcome were assessed with Pearson and Spearman correlation coefficients. Only pupillary bias was positively associated with relapse outcome (Table 1). The Spearman rank order correlation confirmed that the mean pupillary bias correlated with the ordinal outcome of relapse (rho = 0.64, *p* = 0.004). Duration of abstinence did not correlate with relapse outcome (*R* = − 0.17, *p* = 0.49). Desire for alcohol as indicated by DAQ scores trended towards a negative association with relapse outcome and pupillary bias, but was not statistically significant (*R* = − 0.26, *p* = 0.25), (*R* = − 0.41, *p* = 0.09), respectively. Craving rating was significantly associated with OCDS scores (*R* = 0.49, *p* = 0.02) and AUDIT (*R* = 0.67, *p* < 0.001), but not with relapse outcome.

### Prediction of relapse

Pupillary reactions were associated with an ordinal relapse outcome after adjustments of control variables: In the hierarchical logistic regression model, pupillary bias was a significant factor associated with subsequent relapse outcome (*β* = 3.39, *p* = 0.02) which significantly improved the model (LR *χ*^2^ (1) = 9.41, *p* = 0.001). Craving ratings failed to reach significance (*β* = − 1.27, *p* = 0.14) when added in the second step and did not significantly improve the model (LR *χ*^2^ (1) = 2.30, *p* = 0.25). Adding the pupillary diameter before stimulus onset instead did not improve the model (LR *χ*^2^ (1) = 0.08, *p* = 0.78); however, duration of abstinence did (LR *χ*^2^ (1) = 7.18, *p* = 0.007).

Consequently, to adjust for this potential confound, duration of abstinence was entered along with control variables of OCDS, DAQ, AUQ, AUDIT, and BDI, as a first step after which pupillary bias was entered. The variables in this first step explained 47.0% of the variance in relapse outcome 4-months later. Baseline amount of alcohol intake before treatment initiation measured using the AUQ emerged as a significant predictor (*β* = 3.31, *p* = 0.045) while duration of abstinence failed to reach significance (*β* = − 2.40, *p* = 0.059). In the second step, pupillary bias was the only significant predictor (*β* = 3.01, *p* = 0.045) of relapse and explained an additional 26.7% variance in relapse outcome at the 4-month follow-up (LR *χ*^2^ (1) = 5.59, *p* = 0.018; Table 2). The association was in the hypothesized direction, as patients with a stronger degree of pupillary dilation to alcohol images as contrasted with dilation to neutral images had a higher likelihood of relapse. Modeling relapse instead as a binary outcome resulted in similar findings with the first step explaining 42.8% variance and the second step which included pupillary bias explaining an additional 32.0% variance (LR *χ*^2^ (1) = 24.95, *p* < 0.001).

**Table 2:** Hierarchical multiple regression analysis for ordinal regression models of variables predicting relapse outcome at 4-month follow-up

	Std. *β*	Lower 95% CI	Higher 95% CI	*p*	OR
Step 1: change *R*^2^, 0.47 LR *χ*^2^(6) = 11.43					
OCDS	− 0.257	− 2.39	1.92	0.802	0.77
DAQ	− 1.215	− 3.60	0.15	0.186	0.30
AUQ	3.307*	0.77*	7.44*	0.045*	27.3*
AUDIT	− 1.542	− 4.58	0.78	0.224	0.21
BDI	0.023	− 0.15	0.18	0.772	1.02
Duration of abstinence	− 2.410	− 6.04	− 0.54	0.059	0.09
Step 2: change *R*^2^, 0.27 LR *χ*^2^(1) = 5.59*					
OCDS	− 0.128	− 2.82	2.60	0.921	0.88
DAQ	− 0.089	− 2.59	1.75	0.923	0.91
AUQ	3.855	− 0.06	9.56	0.104	47.23
AUDIT	− 1.656	− 5.80	1.41	0.340	0.19
BDI	0.053	− 0.11	0.24	0.529	1.05
Duration of abstinence	− 1.699	− 6.17	1.57	0.361	0.18
Pupillary bias	3.006*	0.48*	6.81*	0.045*	20.22*

*N* = 18. Final model *R*^2^, 0.89 adjusted *R*^2^, 0.74. The *R*^2^ is a log-likelihood-based coefficient of determination (see “Method” for details)

*LR χ*^2^ (*df*), Log likelihood ratio chi-squared test; *df*, degrees of freedom; *p*, probability value; *std. β*, standardized coefficients; *OCDS*, Obsessive Compulsive Drinking Scale; *DAQ*, Desire for Alcohol Questionnaire; *AUQ*, Alcohol Use Questionnaire; *AUDIT*, Alcohol Use Disorder Identification Test; *BDI*, Becks Depression Inventory; *OR*, odds ratio; *CI*, confidence interval

*p<0.05

## Discussion

We show that inter-individual pupillary dilation to alcohol stimuli in alcohol-dependent patients predicts relapse outcome. An objective physiological measure of an increase in pupillary dilation to alcohol stimuli and not a subjective measure of craving predicted the outcomes of no relapse, minor relapse (lasting less than a week), and major relapse (lasting longer than a week). Critically, pupillary reactions significantly and substantially improved the prediction of relapse beyond that achieved by five standardized questionnaires (OCDS, DAQ, AUQ, AUDIT, and BDI) and variance in duration of abstinence. This pilot study lends support to the hypothesis of Tiffany and Carter (1998) who argue that automatic behavioral responses and not subjective craving play a key role in the maintenance of addiction disorders. Craving is often assumed as a defining symptom of addiction and was entered into the DSM-5 and remains part of the International Classification of Diseases (ICD-11) (World Health Organization 1992; Skinner and Aubin 2010; American Psychiatric Association 2013; Murphy et al. 2014; Saunders et al. 2018).

We assessed craving by asking patients to rate the effects of viewing an alcohol image on their temptation to drink. Importantly, the study was designed to circumvent the difficulties of measuring craving in a clinical research setting by asking patients to rate temptation, as a proxy for desire or craving, in an imagined scenario of being an active drinker and outside of the treatment setting. We suggest the measure of craving to be valid as it correlated with standardized questionnaires such as the Obsessive-Compulsive Drinking Scale (OCDS) and prior problematic alcohol use (AUDIT), but crucially, this subjective measure did not correlate with relapse nor did it significantly improve the prediction of relapse. Thus, our findings dovetail with empirical studies reporting correlations between physiological cue-induced reactions and relapse in the absence of a concurrent correlation with subjective craving (Drummond and Glautier 1994; Rohsenow et al. 1994; Grüsser et al. 2004). Notably, studies of stress-induced craving (i.e., craving secondary to a stress challenge), rather than the cue-induced craving under study here, show efficacy as a predictor of relapse (Cooney et al. 1997; Breese et al. 2005; Sinha and Li 2007; Seo et al. 2013). Although the subjective craving measure may be similar, the mechanisms underlying these cue-induced and stress-induced cravings are based on differing theoretical constructs of incentive salience and negative emotionality, respectively (Koob and Volkow 2010). The lack of a correlation between relapse outcome and our craving measure in addition to our control variables of OCDS, DAQ, AUQ, AUDIT, and BDI does not exclude that better self-report measures can provide predictive value of relapse outcome (see Adamson et al. 2009 for review). Moreover, these findings could simply be due to our study being notably underpowered, although the present results suggest a strong link between pupillary reactions and relapse following treatment.

To our knowledge, the present study is the first to observe a prospective predictive relation between differential pupillary dilation and subsequent relapse outcome. This predictive relationship may have clinical value. Pupillary reactions are assumed to occur spontaneously and are considered hard if not impossible to control voluntarily, in particular, the initial reaction (Laeng et al. 2012). In our pupillary waveform analysis, we show that differential pupillary reactions in the 150–250-ms time window correlate with subsequent relapse outcome. This response is within the response time window for neural visual processing, but given its early stage, we are fairly confident that it reflects an implicit response, beyond the influence of the patients’ willingness to react differently to alcohol-related images which requires time for cognitive processing. It has been suggested that the conscious self-knowledge of alcohol craving can serve as a warning signal, which patients can use to seek help or employ self-control strategies to avoid relapse (Heinz et al. 2009). It can therefore be argued that implicit measures are more informative to clinicians as self-awareness and insight into the severity of dependence, a meta-cognitive construct, might be lower in substance-dependent individuals (Goldstein et al. 2009; Cousijn et al. 2011). This is further substantiated by our implicit measures outperforming self-report measures in predicting relapse outcome.

Another clinical application of this measure is as an outcome measure for interventions such as cognitive bias modification or cue-exposure therapy (CET), which directly aims to extinguish learned responses to substance-related stimuli. A recent meta-analysis for CET for AUD shows CET to have no-to-small effect on primary outcomes and a small-to-moderate effect on secondary measures, and notes a lack of methodological quality in the studies reviewed (Mellentin et al. 2017). Differential pupillary reactions to alcohol-related stimuli that are present prior to a CET intervention could potentially act as a moderator of treatment effect and could thus be an important covariate for future studies investigating CET efficacy. As an example, one study which tested a novel attentional training paradigm aimed at decreasing mood reactivity found that baseline pupillary reactions predicted the effect of the intervention (Price et al. 2016). Pupillary responses has similarly been used to predict depression and autism risk in children (Osterling and Dawson 1994; Burkhouse et al. 2015). In a recent study, long- and short-term alcohol abstinent patients showed overactivation of the pupillary response to emotional information (Claisse et al. 2016). This further highlights the potential utility of pupillary responses as a predictor of emotional wellbeing for patients with AUD as well as other patient populations.

There is a general consensus that pupillary reactions to light-controlled stimuli indexes the intensity of the mental activity the subject is currently engaged in and the attentional demands imposed by different tasks or stimuli (Goldinger and Papesh 2012; Laeng et al. 2012). Seminal pupillometry studies reported that the pupils would dilate when an observer viewed positively emotionally valenced stimuli, such as sexually arousing images or political statements consistent with the observer’s interests (Hess et al. 1965; Hess 1965). Similarly, studies have shown increased pupillary dilation when infants view a picture of their own mother as compared with a stranger (Fitzgerald 1968). In a similar vein, numerous contemporary studies demonstrate that pupil dilation reflects reward processing (O’Doherty et al. 2003; O’Doherty et al. 2006; Anderson and Yantis 2012), specifically the sensitivity to reward (Muhammed et al. 2016).

Increased dilation has also been observed for memorization of spans of digits, and arithmetic (Hess and Polt 1964; Beatty and Kahneman 1966; Kahneman 1973) and, thus, expresses the mental effort required by the task. This effect has been shown to be dissociable from emotional reactions (Stanners et al. 1979) and can augment the pupillary dilation attributed to reward prospect (Bijleveld et al. 2009). Indeed, the cognitive account of the pupillary responses as increased “load on attentional capacities” is supported by many studies demonstrating correlations between pupil dilation and increased cognitive load (see Laeng et al. 2012 for review). For instance, the pupillary response is receptive to the congruence effect of the classic Stroop color-naming task, with incongruent words evoking more dilation than congruent words (Laeng et al. 2011).

Pupillary reactions are orchestrated by phasic changes in locus coeruleus (LC) activity, implicated in norepinephrine (NE) activity. The LC innervates brain areas widely known to be involved in selective attention and is believed to play a vital role in fine-tuning the required activity to meet attentional demands (Foote and Morrison 1987). Cognitive demands are by their very nature greater when the environment is uncertain and when surprising events occur, which fits with a model of NE as coding surprise and uncertainty (Dayan and Yu 2006). This interpretation is also in line with more recent evidence relating increased pupillary dilation and thus NE-transmission with uncertainty during decision-making tasks (Preuschoff et al. 2011; Kloosterman et al. 2015; Koenig et al. 2018). Greater reactivity in the LC-NE system to alcohol relative to neutral stimuli could underlie our observation of differential pupil dilation correlating with relapse. One plausible interpretation is that for those patients at greater risk of relapse, greater attentional demands are imposed by alcohol stimuli as they are more unexpected and represent a high degree of uncertainty with respect to the probability of a positive or negative outcome (O’Doherty et al. 2006; Preuschoff et al. 2011).

The present authors are cautious about any strong interpretation of our finding as our study is preliminary, and our design prevents discrimination of the type of cognitive or emotional mechanism involved. The neutral stimuli employed in our study were not matched with alcohol cues on emotional valence. Despite our baseline pupil measures not changing during the course of the neutral trials, further studies may employ a block design rather than interleaving stimulus type to further exclude a contamination effect. Further testing with positive and negatively emotionally valenced stimuli, non-alcohol beverages or in the context of stress-induced craving are required to dissociate emotional valence from arousal effects (Pulido et al. 2010). Previous studies have investigated pupillary dilations and contractions to emotional images of neutral, positive, and negative emotional valence (Bradley et al. 2008; Franzen et al. 2009; Dietz et al. 2011; Vasquez-Rosati et al. 2017). The former two studies find that the pupils generally dilate more to positive and negative stimuli compared to neutral stimuli. In contrast Franzen et al. (2009) and Vasquez-Rosati et al. (2017) show greater pupil dilation to negative images as compared with neutral and positive images. Translating this line of research onto predictors of alcohol relapse will also have to consider the inter-individual variability in the assessment of alcohol as negative or positive. In our present study, we subtracted the pupillary diameter to alcohol-related images as compared with neutral in order to investigate this “bias” in the pupillary dilation towards alcohol as a candidate biomarker of relapse. As such, our approach represents a point of departure from prior studies as it directly assesses the utility of differential pupillary reactions in predicting a clinically relevant outcome. Clearly, more research is required to disentangle the mechanisms underlying our observations, along with further assessment of its consistency in a larger sample size.

The limitations of the study are worth considering. First, the lack of a significant association between self-report measures and relapse outcome could be due to the limited sample size of the study. However, the fact that we show a clear effect of an objective physiological biomarker in such a limited sample argues for a robust effect. Secondly, the length of reported detoxification prior to study participation had considerable variation. Controlling for this confounding variable in the hierarchical regression analysis did not limit the pupillary bias measure in explaining a significant and substantial amount of variance in relapse outcome. However, further studies should assess differential pupillary cue-reactivity and the association to relapse in a sample matched for duration of abstinence. Thirdly, our limited sample prevents us from exploring the relationship between pupillary responses and craving ratings thoroughly. We recommend that further research makes use of multiple trials for each stimulus to avoid missing pupillary samples while employing a craving measure with equal variance across individuals in a larger sample. Finally, our results should be interpreted bearing in mind that we show differential pupillary reactions to have predictive value of relapse at a 4-month follow-up. It is therefore not conclusive that pupillary bias can predict relapse at a 6-month or at 1-year follow-up, as the predictive value of a covariate can change (Miller et al. 1996).

In conclusion, the present results indicate that pupillary dilation to alcohol-related stimuli as compared with neutral stimuli was greater among alcohol dependent patients who subsequently relapsed. Differential pupillary reactions, namely dilation to alcohol stimuli, may be an index of increased attentional demands imposed by the stimuli or of the greater emotional valence attributed to the stimuli. This study shows that the sight of alcoholic beverages, by which alcohol-dependent individuals are daily confronted (e.g., advertisements, supermarkets), evoked a hitherto unknown reaction that was predictive of relapse. Pupillometry presents as a cost-effective and important tool for addiction science in potentially providing robust biomarkers of the vulnerability to future relapse.
